# Interleukin 4 inducible 1 gene (IL4I1) is induced in chicken phagocytes by *Salmonella* Enteritidis infection

**DOI:** 10.1186/s13567-020-00792-y

**Published:** 2020-05-13

**Authors:** Marta Elsheimer-Matulova, Ondrej Polansky, Zuzana Seidlerova, Karolina Varmuzova, Hana Stepanova, Radek Fedr, Ivan Rychlik

**Affiliations:** 1 Veterinary Research Institute, Hudcova 70, 621 00 Brno, Czech Republic; 2 Institute of Biophysics, Academy of Sciences of the Czech Republic, Kralovopolska 135, 612 65 Brno, Czech Republic

## Abstract

In attempt to identify genes that are induced in chickens by *Salmonella* Enteritidis we identified a new highly inducible gene, interleukin 4 induced 1 gene (IL4I1). IL4I1 reached its peak expression (458× induction) in the cecum of newly hatched chickens 4 days post-infection and remained upregulated for an additional 10 days. IL4I1 was expressed and induced in macrophages and granulocytes, both at the mRNA and protein level. IL4I1 was expressed and induced also in CD4 and γδ T-lymphocytes though at a 50-fold lower level than in phagocytes. Expression of IL4I1 was not detected in CD8 T lymphocytes or B lymphocytes. Mutation of IL4I1 in chicken HD11 macrophages did not affect their bactericidal capacity against *S*. Enteritidis but negatively affected their oxidative burst after PMA stimulation. We therefore propose that IL4I1 is not directly involved in bactericidal activity of phagocytes and, instead, it is likely involved in the control of inflammatory response and signaling to T and B lymphocytes.

## Introduction

*Salmonella enterica* serovar Enteritidis (*S.* Enteritidis) is one of the most frequent causes of human gastrointestinal disorders. In the EU, salmonellosis was the second most commonly reported zoonotic infection in humans in 2009 and *S.* Enteritidis was the most frequent serovar [1]. As poultry meat and eggs are the primary causes of human *Salmonella* infections [2], it is believed that *Salmonella* control in poultry will largely prevent these organisms from entering the human food chain [3]. Although both the incidence of human salmonellosis and *Salmonella* prevalence in poultry flocks has nowadays decreased in the EU, *S.* Typhimurium infections in Sub-Saharan Africa has increased [4, 5]. A detailed understanding of *Salmonella*-host interaction is therefore still needed.

Despite the absence of gross clinical signs, newly hatched chickens respond to oral infection with non-typhoid *S.* *enterica* serovars by expressing cytokines such as IL-1β, IL-17 and IFNγ in the cecum [6, 7]. Besides cytokines, many effector genes are induced in the inflamed cecum. The most inducible chicken gene in the cecum in response to *S.* Enteritidis infection seems to be matrix metalloproteinase 7 but chickens also respond to *S.* Enteritidis infection by expression of serum amyloid A, avidin, ExFABP, calprotectin and tens of other genes [8, 9]. Most of the genes and proteins reach their maximal expression in the chicken cecum 4 days post-infection of newly hatched chickens and by 20 days post-infection, the expression of these genes declines back to basal expression levels [8].

When the chickens are infected with the SPI1 mutant *S.* Enteritidis, which is defective in invasion into non-professional phagocytes, most genes induced in the chicken cecum after infection with the wild-type *S.* Enteritidis are hardly induced [8]. This observation can be explained by the absence of *Salmonella* invasion into epithelial cells, absence of exposure of these cells to intracellular LPS, flagella and other pathogen associated molecular patterns (PAMPs) and, consequently, absence of induction of the NF-κB inflammatory signaling pathway [10, 11]. However, this is in contradiction with observations that the *S*. Enteritidis SPI1 mutant is immunogenic in chickens [12, 13]. The *S*. Enteritidis SPI1 mutant therefore must be recognized by the chicken immune system even in the absence of inflammatory signaling.

In this study we therefore compared gene expression in the chicken cecum after infection with the wild-type *S*. Enteritidis and its isogenic SPI1 mutant. We identified several genes during this screening which were inducible by the wild-type *S*. Enteritidis but none specifically induced by the SPI1 mutant only. However, one of the inducible genes was a gene coding for interleukin 4 induced 1 gene (IL4I1, also called LAAO for predicted function L-amino acid oxidase). We did not detect this gene as responding to *S*. Enteritidis infection in our previous studies [8, 14]; nonetheless, this gene turned out to be one of the most inducible chicken genes by *S*. Enteritidis infection. We therefore characterized its expression in the chicken cecum and chicken splenic leukocytes after *S*. Enteritidis infection in detail and tested the role of IL4I1 in *S*. Enteritidis defense in IL4I1 deficient HD11 macrophages.

## Materials and methods

### *Salmonella* Enteritidis infection of chickens

Six male ISA Brown chickens were infected orally at day of hatch with 10^7^ CFU of wild-type *Salmonella* Enteritidis 147 [15] or its isogenic SPI1 mutant [16], and sacrificed 4 days later. Six non-inoculated 5-day-old chickens were included as a control group. Approx. 30 mg of the cecum was collected from each chicken during necropsy, immediately placed into RNAlater (Qiagen) and stored at −80 °C.

In the second experiment, 64 male ISA Brown chickens were infected orally at day of hatch with 10^7^ CFU of wild-type *Salmonella* Enteritidis 147 and sacrificed on day 2, 3, 4, 5, 6, 7, 8, 9, 10, 11, 12, 15, 18, 22, 25 and 29 of life, 4 chickens each day. Sixty-eight non-infected chickens were included as controls; four non-infected chickens were sacrificed on day 1 and the remaining at the same time points as the infected ones. During necropsy, approx. 30 mg of the cecum was collected into RNAlater (Qiagen) and stored at −80 °C.

### Infection of HD11 macrophages

HD11 macrophages were cultivated in RPMI (Lonza) supplemented with 10% fetal calf serum and infected with *S.* Enteritidis 147 at multiplicity of infection (MOI) 1 for 1 h. One hour after the infection, free bacteria were washed away with Dulbecco’s phosphate buffered saline (DPBS, Lonza) and gentamicin was added to fresh RPMI medium (100 μg/mL). One hour later, the medium was replaced with fresh RPMI medium containing 15 µg/mL of gentamicin. Two and twenty-two hours later, i.e. four or twenty-four hours after the infection, cells were washed twice with DPBS, lysed with 1 mL TRI Reagent (MRC) and the lysates were stored at −80 °C. Negative controls included HD11 macrophages treated as the experimental group except for bacterial infection. The experiment was performed in pentaplicates on two independent occasions.

For invasion and multiplication assay, 4 and 24 h after the infection the cells were treated with 0.5% Triton X-100, serially diluted and plated on LB agar plates. For flow cytometry analysis, cells at 4 h after the infection with *S.* Enteritidis 147 pFPV25.1 at MOI 10 were treated with accutase (Sigma) for 10 min, spun at 400 × *g*, washed with DPBS and analyzed by flow cytometry.

### Construction of IL4I1 deficient HD11 macrophages

Inactivation of IL4I1 in HD11 macrophages was performed using Clustered Regularly Interspaced Short Palindromic Repeats (CRISPR)/Cas system. Briefly, custom, all-in-one vector U6gRNA-Cas9-2A-GFP with the target site (underlined) AGG GGGACTCGCCAGCGCCAAGAGG (Sigma-Aldrich) binding to nt 1505–1526 (Gene ID 417039) was transfected into HD11 using Lipofectamine LTX Reagent (Invitrogen). Vector-Lipofectamine LTX complexes were prepared following the manufacturer’s instructions and added to HD11 macrophages. The plate was incubated for 18 h at 37 °C in a CO_2_ incubator. HD11 macrophages were detached from the plate with accutase (Sigma), sorted by FACSAria (BD Biosciences) and GFP-positive cells were cloned by manual dilution. DNA samples, extracted from the clones using DNeasy Blood and Tissue Kit (Qiagen) according to the manufacturer’s instructions, were sequenced by MiSeq System (Illumina). Primers used for the amplification of DNA in PCR with HotStart Taq Plus Master Mix (Qiagen) were CR_T25_Fwd GGAACTCCCTGTTGGGGTTT and CR_T25_Rev GGCAAATGGGTTTGGGGTTC. PCR products were diluted to 0.2 ng/μL and processed using Nextera XT DNA Library Prep Kit (Illumina) following the manufacturer’s manual.

### Reactive oxygen species (ROS) detection in HD11 macrophages

Three different IL4I1^−/−^ macrophage clones after stimulation with phorbol 12-myristate 13-acetate (PMA) were used to detect reactive oxygen species in HD11 IL4I1^+/+^ with L-012 chemiluminescence probe (Wako Chemicals). Briefly, cells in a 96-well plate were washed with warm Hank’s Balanced Salt Solution (HBSS, Lonza). Fresh HBSS containing 8-amino-5-chloro-7-phenylpyridopyridazine and L-012 (20 µM) was added to wells and right before placing the plate in a microplate reader Synergy 2 (BioTek Instruments), PMA (Sigma-Aldrich) was added to wells to a final concentration 10 µg/mL. Experiment was performed in five replicates per experimental group and two wells per group were used as negative controls without PMA stimulation. Luminescence was measured for 2 h at 37 °C.

### Fluorescence-activated cell sorting

Splenic leukocytes were sorted by fluorescence activated cell sorting in two independent experiments. In each experiment, three 42-day-old chickens were intravenously infected with 10^7^ CFU of wild-type *Salmonella* Enteritidis 147 and sacrificed 4 days later. Three non-infected chickens were included as controls in both experiments. Leukocytes from the spleen were isolated as described earlier [17]. In total 4 × 10^7^ cells from each sample were used for surface marker staining. The first panel of primary antibodies (all Southern Biotech, Alabama, USA) consisted of anti-CD45:APC (clone LT40), anti-CD4:FITC (clone CT-4), anti-CD8α:SPRD (clone CT-8) and anti-TCR1:PE (clone TCR-1). The second panel of antibodies consisted of anti-CD45:APC (clone LT40), anti-monocyte/macrophage:FITC (clone KUL01) and anti-Bu-1:PE (clone AV20). The samples were sorted by a BD FACSAria II operated by Diva software (BD Biosciences) with nozzle size set to 85 µm, sheath pressure 45 psi, frequency 47 kHz and four-way purity sort mask. The number of sorted cells ranged from 0.3–2 × 10^6^ depending on the abundance of the leukocyte subpopulation in the analyzed samples.

The purity of the sorted subpopulation was re-tested by flow cytometry comparing positive staining for specific antigens to all CD45 positive cells. The purity of CD8+ T-lymphocytes sorted in the first experiment was 96.7 ± 1.4 (mean % ± SD), CD4+ T-lymphocytes 94.1 ± 2.1, γδ T-lymphocytes 93.5 ± 2.6, B-lymphocytes 92.4 ± 3.1 and monocytes/macrophages 89.9 ± 3.0. Purity of CD8 + T-lymphocytes sorted in the second experiment was 96.8 ± 1.3, CD4+ T-lymphocytes 94.7 ± 1.9, γδ T-lymphocytes 97.0 ± 1.2, B-lymphocytes 93.2 ± 4.1, monocytes/macrophages 95.6 ± 2.1 and granulocytes 81.8 ± 12.0. Granulocytes were only sorted in the second experiment based on their FSC/SSC characteristics.

### RNA and protein purification

Ceca of infected and non-infected chickens, HD11 cells or sorted leukocyte subpopulations were used for parallel protein and RNA isolation. The samples were recovered from RNAlater storage, mixed with 1 mL of TRI Reagent (MRC) and homogenized with MagNA Lyser (Roche). Fifty μL of bromoanisole was added to the homogenate and after centrifugation at 14 000 × *g* for 15 min, the upper phase containing RNA was collected and purified with RNeasy Mini Kit (Qiagen). One μg of RNA was immediately reverse transcribed using oligo(dT) primers and M-MLV reverse transcriptase (Invitrogen). cDNA was diluted 10 × and stored at −20 °C. Proteins captured in the lower phenolic phase were precipitated with acetone according to the manufacturer’s recommendation (MRC).

### Protein identification by Oribtrap Velos Pro mass spectrometry

Protein pellets were processed according to the modified filter-aided sample preparation (FASP) method [18] using a Vivacon 500 device with MWCO of 10 kDa (Sartorius Stedim Biotech) as described earlier [19]. Tryptic peptides were labeled by the stable isotopes using dimethyl labeling method [20]. Peptides from control samples were labeled with CH_2_O and NaBH_3_CN (light tag) and peptides from experimental group were labeled with CD_2_O and NaBH_3_CN (medium tag). Samples were mixed at a 1:1 ratio and analyzed in 3 independent LC–MS/MS runs using the Dionex UltiMate 3000 RSLC nano system connected to an Orbitrap Velos Pro mass spectrometer (Thermo Scientific). Data were analyzed using the Proteome Discoverer v.1.4. MS/MS spectra identification was performed by SEQUEST searching against the *Gallus gallus* Uniprot database released on September 9, 2013. Only peptides with a false discovery rate (FDR) < 1% were included in semiquantitative analysis which was based on ratios of peptide peak areas.

### Protein quantification by triple quadrupole mass spectrometry

Quantitative protein mass spectrometry was based on selected-reaction monitoring (SRM) using nanoLC Ultimate 3000 RSLC (Dionex) and triple quadrupole mass spectrometer TSQ Vantage (Thermo Scientific). TSQ Vantage was optimized for sensitivity at the expense of resolution and selectivity—for both precursor (Q1) and fragment (Q3) ion selection, the peak width at half maximum was 1.5 Da. For ionization, 1000 V spray voltage at 325 °C capillary temperature was used. The collision pressure was set to 1.2 mTorr of argon. Pinpoint software v.1.2 (Thermo Scientific) was used for SRM development. For both GAPDH and IL4I1, one unique peptide and 3 transitions, i.e. peptide-fragment m/z pairs, per each peptide were selected. As high resolution and accurate mass is absent on TSQ, peptide identification was based on precise co-elution with spiked-in isotopically labeled standard (PEPotec SRM Peptide Library, Grade 1, Thermo Scientific) and on fragmentational pattern of labeled and native peptide pairs. For peptide sequences, fragment ion types, peptide and fragment mass-to-charge ratios and optimal collision energies see Additional file 1.

### Quantitative real-time PCR

Quantitative real-time PCR was used for the verification of proteomic data from chicken ceca and for quantification of IL4I1 mRNA/cDNA in HD11 macrophages and sorted leukocyte subpopulations. Real-time PCR was performed in duplicates with QuantiTect SYBR Green PCR Master Mix (Qiagen) as described earlier [8]. Primers are listed in the Additional file 2. The Ct values of the genes of interest were normalized to an average Ct value of three house-keeping genes GAPDH, TBP and UB, and the relative expression of each gene of interest was calculated as 2^−ΔCt^.

## Results

### Proteins induced in the chicken cecum after infection with *S*. Enteritidis

**Figure 1:**
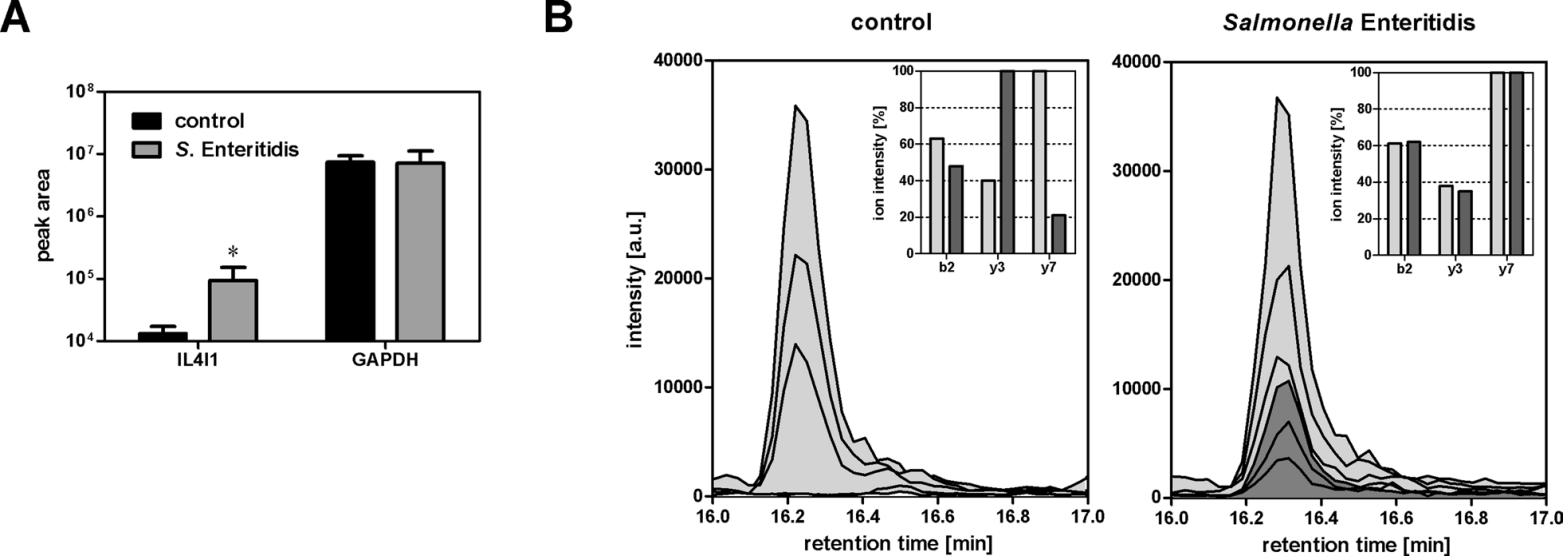
**IL4I1 protein expression in the chicken cecum after*S.*** **Enteritidis infection determined by triple quadrupole mass spectrometry analysis of proteins from the ceca of control 5-day-old chickens and age-matched experimental chickens 4** **days post-infection with*S*. Enteritidis. A** IL4I1 and GAPDH protein quantity expressed as mean peak area of a specific peptide ± SD. Significantly higher peak area was recorded only for IL4I1 and not for housekeeping GAPDH protein. Asterisks—statistically significant difference from non-infected chickens (Mann–Whitney U test, *P* ≤ 0.05). **B** Targeted quantification of IL4I1 protein. SRM chromatogram for three transitions of one of IL4I1 peptide from representative cecal sample of non-infected (left panel) or infected (right panel) chicken. Light gray, chromatogram of synthetic peptide standard; dark gray, chromatogram of peptide from cecal samples. The bar charts in top-right corners present comparison of SRM transition intensity ratios. In the case of positive identification, relative intensities should be identical for synthetic heavy and native light peptide, as can be seen in the right panel but not left panel since IL4I1 was not detected in the ceca of control, non-infected chickens.

Fourteen proteins were significantly induced in chickens infected with wild-type *S*. Enteritidis in comparison to the SPI1 mutant infected or non-infected chickens (Additional file 2). On the other hand, not a single protein was expressed at significantly higher levels in chickens infected with the SPI1 mutant in comparison to chickens infected with wild-type *S*. Enteritidis. Thirteen out of fourteen proteins induced by wild-type *S*. Enteritidis were identified in our previous studies [8, 9, 14]. However, there was a new protein, IL4I1, which we identified as responsive to *S*. Enteritidis infection for the first time. Since Orbitrap VelosPro mass spectrometry used in the initial screening provides only semiquantitative information, in the next step we determined the expression of IL4I1 protein by triple quadrupole protein mass spectrometry. This analysis showed that the IL4I1 protein was induced more than 7× in the cecum after *S*. Enteritidis infection (Figure 1). Finally, when we used real-time PCR for the verification of IL4I1 mRNA expression in the same samples as used for protein mass spectrometry, IL4I1 exhibited a 458× induction fold induction in the chicken cecum after the infection with wild-type *S*. Enteritidis.

### Expression of IL4I1 in the cecum after oral infection of newly hatched chickens

**Figure 2:**
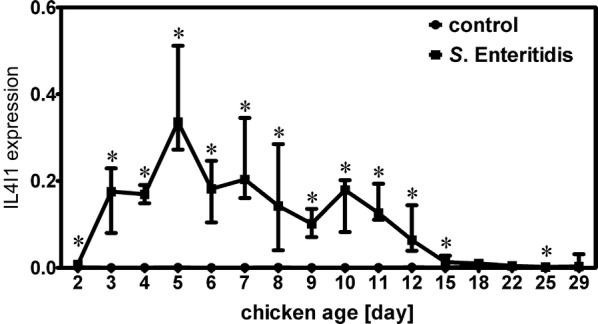
**Course of IL4I1 mRNA expression in the chicken cecum after*S***. **Enteritidis infection determined by real-time PCR.** Chickens were infected at the day of hatch, sacrificed at time points as indicated and expression of IL4I1 was determined by reverse transcribed real time PCR. On days 3, 6, 9, 10 and 29, absolute Ct values for IL4I1 were below a value 30 in 2 out 4 tested non-infected chickens, and on days 11 and 12 absolute Ct values for IL4I1 were below 30 in 1 out 4 non-infected chickens indicating low IL4I expression in the cecum in the absence of infection. Asterisks—statistically significant differences from non-infected chickens (Mann–Whitney U test, *P* ≤ 0.05).

In the next experiment we verified and determined the course of IL4I1 expression in the cecum of *S*. Enteritidis infected chickens by real-time PCR using the samples from our previous study [8]. At mRNA level, IL4I1 was maximally expressed in the cecum 4 days after the infection. Three, four and five days post-infection, IL4I1 exhibited 400–500 fold induction, which makes this gene one of the most inducible gene in the chicken cecum after *S*. Enteritidis infection [8]. Its expression decreased back to the basal level around day 18 of life (Figure 2) corresponding to decreasing *S*. Enteritidis counts in the cecum, liver and spleen [8].

### Expression of IL4I1 in chicken leukocytes

**Figure 3:**
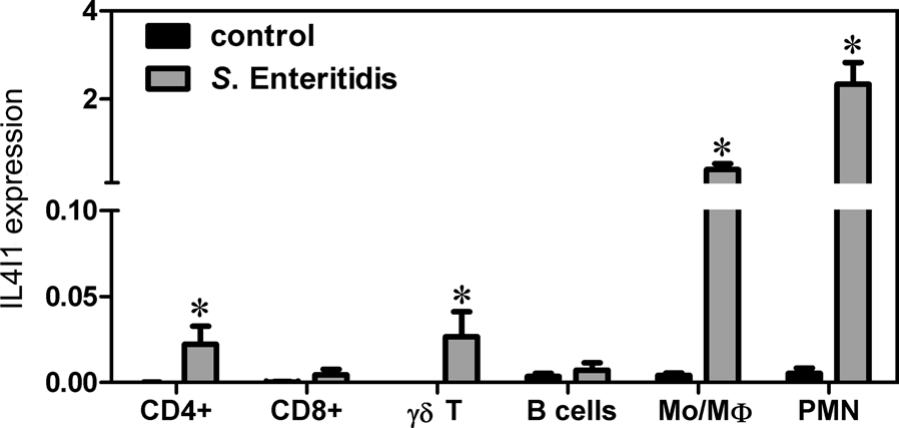
**IL4I1 mRNA expression in chicken leukocyte sub-populations after*S***. **Enteritidis infection determined by real-time PCR.** Asterisks indicate statistically significant differences from the control (non-infected) group (Kruskal–Wallis test followed by post hoc Dunn’s test, *P* ≤ 0.05). Samples are marked as CD4 T-lymphocytes, CD8 T-lymphocytes, γδ T-lymphocytes, B-lymphocytes, monocytes/macrophages and granulocytes (PMN—polymorphonuclear cells).

IL4I1 has previously been detected in human macrophages and B lymphocytes [21]. Next we therefore tested the expression of IL4I1 in chicken macrophages, granulocytes, B-lymphocytes, and CD8+ , CD4+ and γδ T-lymphocytes purified by flow cytometry from the spleen of chickens infected with *S*. Enteritidis. To obtain enough leukocytes for cell sorting, this experiment was performed with 42 days old chickens, unlike the previous experiments performed with chickens after hatching. IL4I1 transcripts increased 103-, 448-, 237- and 102-fold after *S*. Enteritidis infection in macrophages, granulocytes, CD4 + and γδ T-lymphocytes, respectively (Figure 3), though absolute expression levels of IL4I1 in CD4+ and γδ T-lymphocytes were significantly lower than in macrophages and granulocytes (Figure 3). Macrophages and granulocytes therefore represented leukocyte subpopulations with the highest IL4I1 expression.

**Figure 4:**
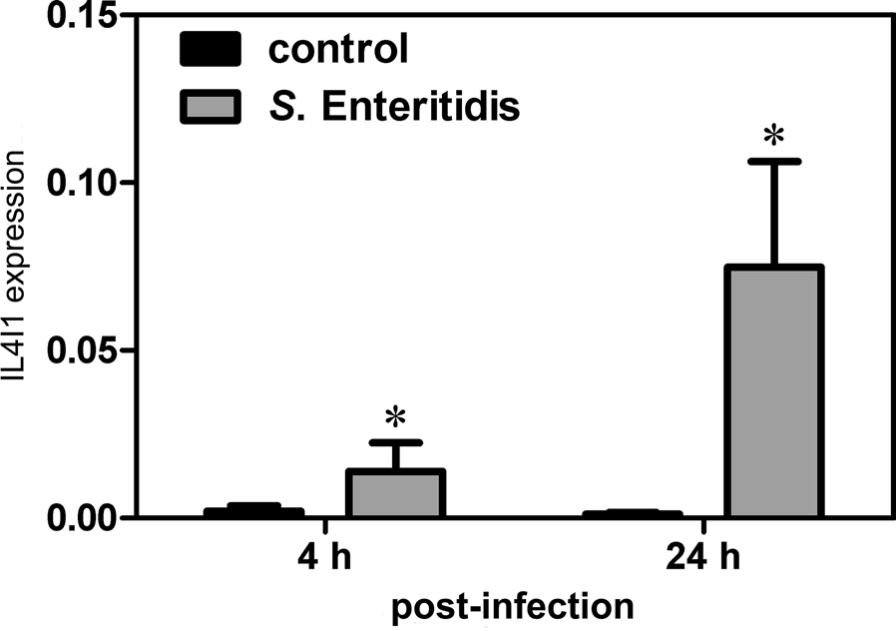
**IL4I1 mRNA expression in HD11 macrophages 4 and 24** **h post-infection with*S***. **Enteritidis.** Asterisks—statistically significant differences from the control (non-infected) macrophages (Kruskal–Wallis test followed by post hoc Dunn’s test, *P* ≤ 0.05).

Due to the IL4I1 expression in chicken macrophages in vivo, its expression was tested also in HD11 chicken macrophage-like cell line. Real-time PCR showed that IL4I1 expression in HD11 macrophages increased 5.42- and 40.72-fold 4 and 24 h after infection with wild-type *S.* Enteritidis, respectively (Figure 4).

### Oxidative burst in HD11 macrophages

**Figure 5:**
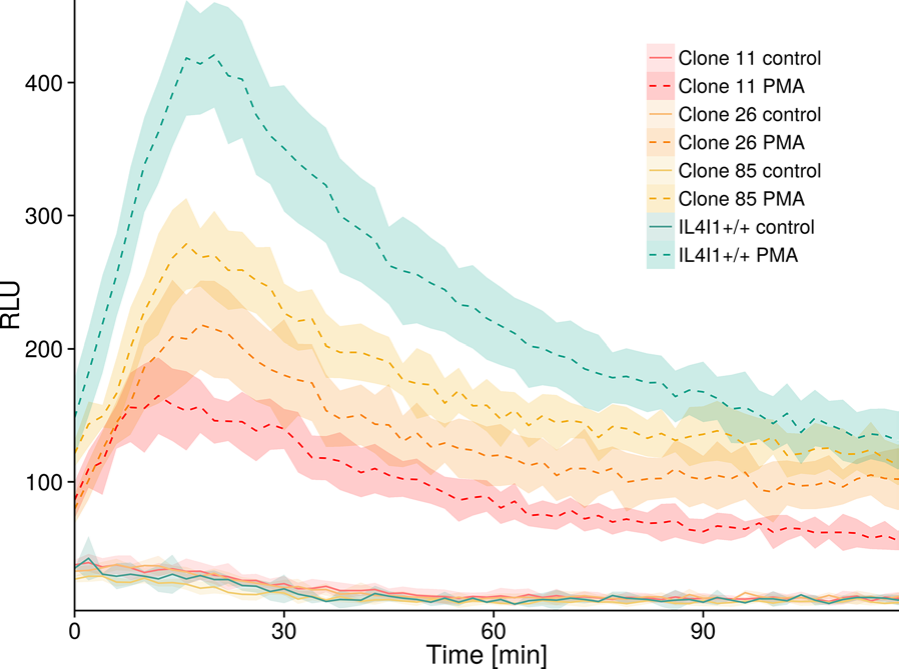
**Oxidative burst in IL4I1-deficient macrophages after stimulation with PMA as measured with a chemiluminescent probe L-012.** Lines represent average values from pentaplicates for each time point over 2 h and shaded ribbons surrounding lines represent 95% confidence intervals.

Predicted function of IL4I1 is conversion of L-amino acids and water into ketoacids, ammonia and H_2_O_2_ [22–24]. Next we therefore constructed IL4I1-deficient HD11 macrophages using CRISPR/CAS system and determined oxidative burst following stimulation with PMA in wild-type HD11 cell line and three IL4I1-deficient HD11 clones (for nucleotide sequences of the CRISPR/Cas target region in HD11 IL4I1^+/+^ cells and IL4I1^−/−^ clones, see Additional file 3). Significant increase in oxidative burst was detected in both wild-type and IL4I1^−/−^ HD11 macrophages when compared to non-stimulated control macrophages. However, IL4I1^−/−^ macrophages produced significantly lower levels of ROS than IL4I1^+/+^ macrophages from the 4^th^ minute of the experiment up to 86^th^ minute of the experiment (Figure 5).

### Invasion and multiplication of Salmonella in IL4I1-deficient macrophages

**Figure 6:**
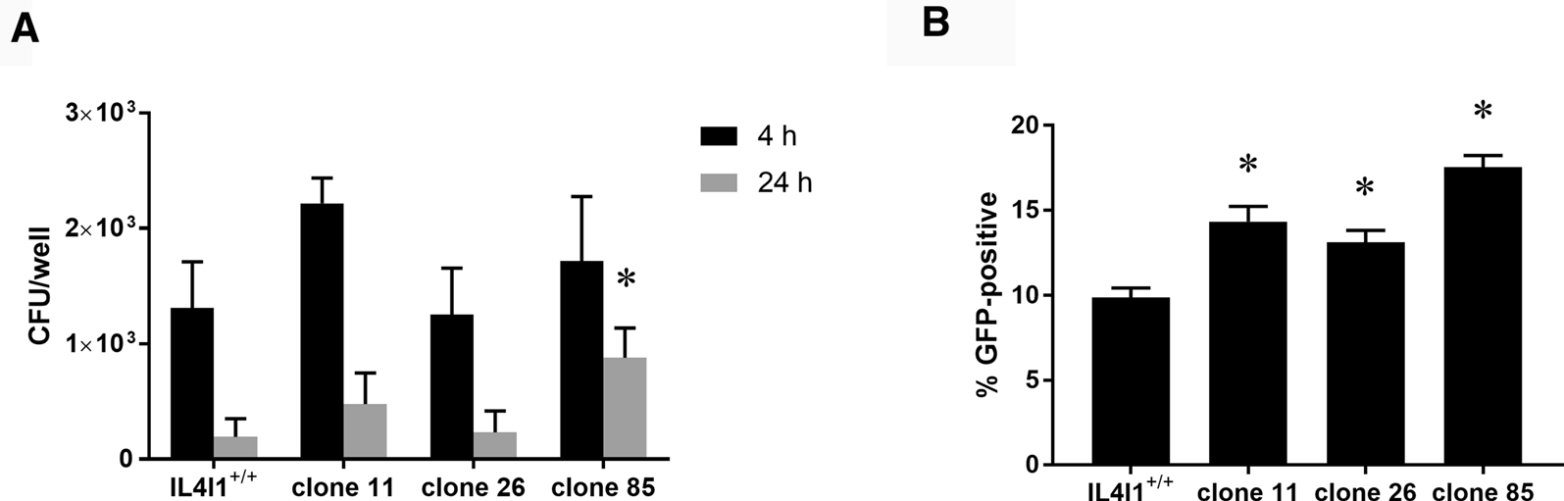
**Invasion and multiplication of*S*. Enteritidis in IL4I1-deficient macrophages. A** Using gentamicin protection assay, with a single exception (clone 85 at 24 h compared with IL4I1^+/+^ macrophages), IL4I1^+/+^ and IL4I1^−/−^ macrophages did not show any difference in killing activity of *S*. Enteritidis. **B** Association of GFP expressing *S*. Enteritidis with HD11 macrophages 4 h after the infection. More IL4I1^−/−^ macrophages were associated with *S*. Enteritidis in comparison to IL4I1^+/+^ macrophages. Asterisks—significantly different from the wild type IL4I1^+/+^ macrophages. Data both in **A** and **B** originate from a single assay.

The lower capacity of ROS production in IL4I1^−/−^ macrophages could result in impaired *Salmonella* killing. Gentamicin protection assay, however, did not show any differences in killing activity of wild-type IL4I1^+/+^ and IL4I1^−/−^ macrophages after the infection with *S*. Enteritidis except for one clone out of three 4 h post-infection (Figure 6A). Next we infected macrophages with *S*. Enteritidis constantly expressing GFP and determined percentage of GFP^+^ HD11 macrophages 4 h after the infection. In this case, significantly more IL4I1^−/−^ macrophages (14 ± 0.6%, 13 ± 0.5%, and 18 ± 0.5%) were infected with *S*. Enteritidis than IL4I1^+/+^ macrophages (10 ± 0.4%) (Figure 6B).

## Discussion

In this study we identified a new gene which has not been associated with chicken response to *S*. Enteritidis infection so far. This gene, interleukin 4 inducible 1 gene (IL4I1), was induced more than 400-fold at transcriptional level, which made it the second most inducible gene after matrix metalloproteinase 7 [8]. To exclude any doubts, we confirmed its induction at transcriptional level by real-time PCR with additional two primer pairs specific to different parts of IL4I1 mRNA with the same results (alternative primer pairs and target exons are listed in Additional file 2). Transcription of IL4I1 in the chicken cecum after oral infection of newly hatched chicken was similar to that of the majority of inducible genes, i.e. it reached its peak 4 days post-infection and then gradually decreased back to basal expression level [8]. IL4I1 has been already identified in chicken macrophages or fibroblasts as responding to avian leukosis virus infection [25, 26]. When we retrospectively looked at our previous reports, IL4I1 was detected as inducible in the caecum of 1 out of 3 chickens infected with *S*. Enteritidis (and therefore excluded from further considerations) [8] and in the liver after intravenous infection with *S*. Enteritidis [27].

Expression of IL4I1 was recorded in human macrophages, dendritic cells and B-lymphocytes [21] or Th17 CD4 T lymphocytes [28]. We therefore tested IL4I1 expression in chicken splenic leukocytes. In 42-day-old chickens, i.e. chickens of different age and possibly different composition of splenic leukocytes than chickens used for oral inoculation, IL4I1 was expressed in macrophages, granulocytes, CD4 + and γδ T-lymphocytes though the expression in both T-lymphocytes subpopulations, in comparison with phagocytes, was quite low. We did not detect IL4I1 in chicken B lymphocytes or CD8 T lymphocytes. High expression of IL4I1 in phagocytes indicated that IL4I1 might be important for pathogen inactivation in phagocytes. However, when we inactivated IL4I1 gene in HD11 macrophages, no differences in intracellular proliferation of *S*. Enteritidis were detected using gentamicin protection assay and only a minor increase in *Salmonella*-positive IL4I1^−/−^ macrophages when compared to IL4I1^+/+^ macrophages was observed using flow cytometry. This may corroborate with lower oxidative burst detected in IL4I1^−/−^ macrophages. Nevertheless, these differences were quite low and the inability of ROS produced by IL4I1 in macrophages to inactivate *Salmonella* is in agreement with in vitro studies performed with LAAOs from snake venom, which inhibited growth of *Staphylococcus*, *Pseudomonas* or *Acinetobacter*, but did not show any effect on *E. coli*, a close relative to *Salmonella* [29, 30]. The minor difference in the bactericidal effect of IL4I1^+/+^ and IL4I1^−/−^ macrophages rather supports a hypothesis on hydrogen peroxide acting as an attractant and activating molecule for other leukocytes [31–33] although we cannot exclude that IL4I1 in a permanent cell line may not behave as in native leukocytes.

In this study we have shown that IL4I1 is one of the most inducible genes in the chicken cecum after infection with *S*. Enteritidis. However, its function is not specific to *Salmonella* infection since it was induced in primary chicken cell lines also after infection with avian leucosis virus [25, 26]. This gene is highly inducible in phagocytes, i.e. macrophages and granulocytes and to a lesser extent in CD4 and γδ T lymphocytes. It was not expressed in CD8 T lymphocytes or B lymphocytes. Based on the experiments with IL4I1-deficient HD11 macrophages, it seems that IL4I1 is not directly involved in pathogen inactivation via ROS production but more likely its function is to regulate T or B lymphocytes, as proposed earlier [34, 35].

## Supplementary information

**Additional file 1. Peptide sequences, fragment ion types, peptide and fragment mass-to-charge ratios and optimal collision energy in targeted LC–MS analysis.**

**Additional file 2. List of genes identified in LC–MS screening, and primers and results from real-time PCR.**

**Additional file 3. 3 Nucleotide sequences of the CRISPR/Cas target region in HD11 IL4I1 +/+ cells and IL4I1-/- clones.**
