# Supplementary material for: Interleukin 4 inducible 1 gene (IL4I1) is induced in chicken phagocytes by Salmonella Enteritidis infection
Source: Vet Res. 2020 May 13;51:67. doi: 10.1186/s13567-020-00792-y (PMC7222322; doi:10.1186/s13567-020-00792-y)
Supplement: Supplementary file 3 — Additional file 3. 3 Nucleotide sequences of the CRISPR/Cas target region in HD11 IL4I1 +/+ cells and IL4I1-/- clones. [file 13567_2020_792_MOESM3_ESM.pdf]

Additional File 3. Nucleotide sequences of the CRISPR/Cas target region in HD11 IL4I1+/+ cells and IL4I1-/- clones.

cells	1501					mutation
**HD11 IL4I1$^{+/+}$**	T A C T G G G A C T	C G C C A G C G – C	C A A G A G G T T C	T C G T G C T T C C	C T G A G T A T T G	
	T A C T G G G A C T	C G C C A G C G – C	C A A G A G G T T C	T C G T G C T T C C	C T G A G T A T T G	
**clone #11**	T A C T G G G A C T	C G C C A G C G C C	C A A G A G G T T C	T C G T G C T T C C	C T G A G T A T T G	frameshift insertion
	T A C T G G G A C T	C G C C A G C G – C	– – – – – – – – – –	– – – – – – – – – –	– – – – – T A T T G	frameshift deletion
**clone #26**	T A C T G G G A C T	C G C C A – – G – C	C A A G A G G T T C	T C G T G C T T C C	C T G A G T A T T G	frameshift deletion
	T A C T G G G A C T	C G C C A – – – – –	– – – – – – – – – –	– – – C G C T T C C	C T G A G T A T T G	frameshift deletion, substitution
**clone #85**	T A C T G G G A C T	C – – – – – – – – –	– – – – – – – – – –	– – – – – – – – – –	– – – – – – – – – G	frameshift deletion
	T A C T G G G A C T	C G C C A G C G C C	C A A G A G G T T C	T C G T G C T T C C	C T G A G T A T T G	frameshift insertion
